# Higher genotypic diversity and distinct assembly mechanism of free-living Symbiodiniaceae assemblages than sympatric coral-endosymbiotic assemblages in a tropical coral reef

**DOI:** 10.1128/spectrum.00514-24

**Published:** 2024-06-14

**Authors:** Sitong Lin, Ling Li, Zhi Zhou, Huatao Yuan, Osama S. Saad, Jia Tang, Wenqi Cai, Kefu Yu, Senjie Lin

**Affiliations:** 1 State Key Laboratory of Marine Environmental Science, College of Ocean and Earth Sciences, Xiamen University, Xiamen, China; 2 School of Marine Science and Engineering, Hainan University, Haikou, China; 3 School of Marine Sciences, Guangxi University, Nanning, China; 4 Department of Marine Sciences, University of Connecticut, Groton, Connecticut, USA; Universitetet i Oslo, Oslo, Norway

**Keywords:** *in hospite *Symbiodiniaceae, *ex hospite *Symbiodiniaceae, ITS2, diversity, deterministic processes, stochastic processes

## Abstract

While *in hospite* Symbiodiniaceae dinoflagellates are essential for coral health, ambient free-living counterparts are crucial for coral recruitment and resilience. Comparing free-living and *in hospite* Symbiodiniaceae communities can potentially provide insights into endosymbiont acquisition and recurrent recruitment in bleaching recovery. In this study, we studied coral-endosymbiotic and ambient free-living Symbiodiniaceae communities in the South China Sea. We collected samples from 183 coral and ambient plankton samples and conducted metabarcoding to investigate the diversity distribution, driving factors, and assembly mechanisms of the two groups of Symbiodiniaceae. Results revealed *Cladocopium* C1 and *Durusdinium* D1 as dominant genotypes. We detected a higher genotypic diversity in free-living than *in hospite* symbiodiniacean communities, but with shared dominant genotypes. This indicates a genetically diverse pool of Symbiodiniaceae available for recruitment by corals. Strikingly, we found that the cooler area had more Symbiodiniaceae thermosensitive genotypes, whereas the warmer area had more Symbiodiniaceae thermotolerant genotypes. Furthermore, *in hospite* and free-living Symbiodiniaceae communities were similarly affected by environmental factors, but shaped by different assembly mechanisms. The *in hospite* communities were controlled mainly by deterministic processes, whereas the ambient communities by stochastic processes. This study sheds light on the genetic diversity of source environmental Symbiodiniaceae and differential assembly mechanisms influencing Symbiodiniaceae inside and outside corals.

**IMPORTANCE:**

Symbiodiniaceae dinoflagellates play a pivotal role as key primary producers within coral reef ecosystems. Coral-endosymbiotic Symbiodiniaceae communities have been extensively studied, but relatively little work has been reported on the free-living Symbiodiniaceae community. Conducting a comparative analysis between sympatric coral-endosymbiotic and free-living Symbiodiniaceae communities can potentially enhance the understanding of how endosymbiont communities change in response to changing environments and the mechanisms driving these changes. Our findings shed light on the genetic diversity of source environmental Symbiodiniaceae and differential assembly mechanisms shaping free-living and *in hospite* Symbiodiniaceae communities, with implications in evaluating the adaptive and resilient capacity of corals in response to future climate change.

## INTRODUCTION

Despite their occurrence in oligotrophic waters, coral reefs are productive and economically important tropical marine ecosystems that support high biodiversity (1, 2). Corals’ ecological success is mainly driven by the mutualistic endosymbiosis between scleractinian corals and dinoflagellates of the family Symbiodiniaceae (3, 4). Symbiodiniacean dinoflagellates are classified into 16 lineages, with 11 described genera and five yet-to-be formally described clades (3, 5–7). Symbiodiniaceae have enormous genetic diversity and physiological and ecological divergence, with hundreds of recognized distinct genotypes (3, 8–10). Symbiodiniaceae in general are key primary producers in marine ecosystems (11, 12), both as endosymbionts and as free-living plankton. Symbiodiniacean dinoflagellates form symbiotic relationships with numerous marine invertebrates and protist hosts, including foraminifera, sponges, mollusks, ciliates, and scleractinian corals (13–18). Free-living Symbiodiniaceae include facultative and obligate free-living types, and can inhabit water columns, sediments, macroalgal beds, and fish feces (18–21). There are also marked geographic variations in the Symbiodiniaceae community structure (22).

The facultative free-living Symbiodiniaceae are important for endosymbiont recruitment for many scleractinian corals. In general, some corals inherit symbiodiniacean symbionts from parents (i.e., vertical transmission), whereas others acquire symbionts from the ambient environment (i.e., horizontal transmission). Vertical transmission maintains higher genetic fidelity from generation to generation (23, 24), while horizontal transmission allows the recruitment of new symbionts (25). Corals can switch or shuffle symbionts in response to stress such as climate warming (26–29). For instance, *Acropora millepora* (Ehrenberg, 1834) can change its dominant endosymbiont from *Cladocopium* ITS2 type C2 to more thermotolerant *Durusdinium* spp. in response to increased temperature during a natural bleaching event (26), which can increase the temperature tolerance of corals by approximately 1–1.5°C (29). Therefore, free-living Symbiodiniaceae communities potentially serve as reservoirs of endosymbionts for corals (26, 30) and may play a crucial role in coral recruitment and resilience (31–34).

Coral-endosymbiotic Symbiodiniaceae diversity has been extensively studied, but relatively little work has been reported on the diversity and distribution of the free-living Symbiodiniaceae community. Fujise et al. (21) found that the free-living Symbiodiniaceae community on Heron Island in the Great Barrier Reef had higher genetic disparity at the genus level than scleractinian coral symbionts, and there was an overlap between them. This is consistent with the tenet of population genetics that a source population holds a higher genetic diversity than end populations (35). Therefore, we hypothesize that ambient Symbiodiniaceae community has higher richness and diversity than, but same dominant genotypes as, *in hospite* assemblage. Moreover, the immediate habitat conditions inside corals and ambient environment are different, but what factors shape the *in hospite* and ambient free-living Symbiodiniaceae communities is not well understood and is understudied. Previous studies have shown that the Symbiodiniaceae communities in corals are mainly influenced by temperature and salinity (36, 37). Free-living Symbiodiniaceae in the water column adjacent to corals also live in the same environment and potentially are influenced by the same environmental factors. Based on this, we hypothesize that environmental factors have similar effects on *in hospite* and ambient Symbiodiniaceae communities. In addition, the assembly mechanisms that shape Symbiodiniaceae community structure have not been studied for *in hospite* or free-living Symbiodiniaceae. Understanding the similarity and distinction in the mechanisms has significant implications for understanding the establishment and breakdown of the coral-Symbiodiniaceae mutualism and for restoration of degraded corals. Neutral-based stochastic and niche-based deterministic processes have been shown to play a crucial role in the assembly of microbial and phytoplankton communities (38, 39). These processes are thought to jointly regulate the structure of microbial communities, and their relative influence can vary with changing environmental conditions (40, 41). Stochastic processes, including stochastic immigration, emigration, and dispersal events, can randomly alter species composition patterns (42). In contrast, deterministic processes including biotic interactions, environmental filtering, and dispersal-related factors can shape species composition and diversity (43). We posit that different assembly mechanisms dominate the assembly of *in hospite* and ambient free-living Symbiodiniaceae communities.

To examine these three hypotheses, a study was conducted to investigate the diversity of both *in hospite* and planktonic free-living Symbiodiniaceae, the latter of which includes transiently or permanently free-living species. Our study sites included two large coral reef ecosystems in the South China Sea (SCS), one off Hainan Island, a major island with a large human population, and the other off Xisha Archipelago, a remote and undeveloped land, both of which feature high biodiversity (44–46). We focused on two coral species, *Pocillopora damicornis* (47) and *Galaxea fascicularis* (48), which are widespread scleractinian corals in the Pacific Ocean. They also represent two different ecotypes and symbiont acquisition modes. *P. damicornis* is sensitive to environmental changes and inherits symbionts vertically (49), whereas *G. fascicularis* is a bleaching-resistant species and recruits symbionts via horizontal transmission (49, 50). We used next-generation sequencing (NGS) and comparative analyses to investigate the distribution, diversity, driving factors, and assembly mechanisms of the Symbiodiniaceae communities in these two scleractinian coral species (114 specimens) and coral surrounding water samples (69 samples). The results shed light on the dominant assembly mechanism and influencing factors in the two Symbiodiniaceae communities and provide a new perspective for evaluating the adaptive and resilient capacity of corals in response to future climate change.

## MATERIALS AND METHODS

### Sampling regions and sample collection

**Fig 1:**
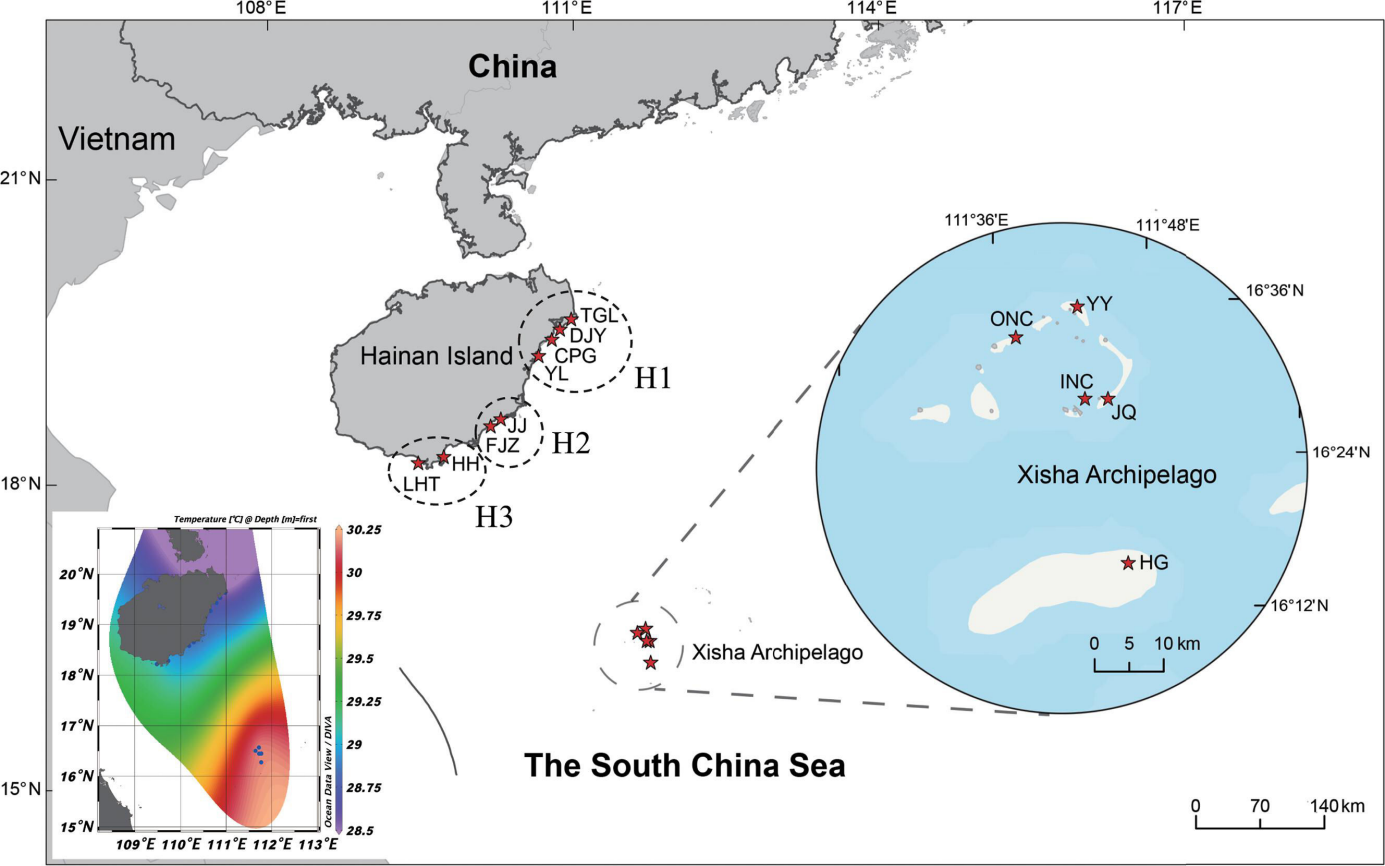
Sampling sites in the two major study areas (Hainan Island and Xisha Archipelago) in the northern South China Sea. H1 includes four stations: TGL (Tongguling), DJY (Dongjiao Yelin), CPG (Changpigang), and YL (Yinglong Bay); H2 consists of two stations: JJ (Jiajing Island) and FJZ (Fenjiezhou Island); H3 comprises two stations: HH (Houhai) and LHT (Luhuitou). YY, Yinyu Island; JQ, Jinqing Island; HG, Huaguang Reef; INC, inner Yongle atoll; ONC, outer Yongle atoll. The lower left plot shows the sea surface temperature at each station.

Corals and coral-surrounding ambient water were sampled from two regions in the northern South China Sea, Hainan Island (18.217481°N, 109.483781°E to 19.628239°N, 110.983908°E) and Xisha Archipelago (16.260556°N, 111.627778°E to 16.580278°N, 111.778889°E), representing coastal and oceanic environments, respectively (Fig. 1; Table S1). In total, 114 coral samples (63 colonies of *G. fascicularis* and 51 colonies of *P. damicornis*) and 69 seawater samples were collected. Of these were 46 coral samples and 24 water samples collected from eight Hainan Island sampling stations in September 2019, and 68 coral samples and 45 water samples collected from five Xisha Archipelago sampling stations in August–September 2020. The coral samples were collected from depths of 1–20 m, and the seawater samples were collected from depths of 1–50 m.

At each sampling station, a small piece (~1 cm^2^) from each healthy coral colony was collected using a hammer and chisel during scuba diving, following the regulations of the Hainan Province government. The coral pieces were immediately washed with filtered seawater. Seawater within a few centimeters surrounding the surfaces of the coral colonies was also collected, with 0.5 L of each sample at Hainan Island and 15 L per sample at the Xisha Archipelago sites, and was filtered through a 200-µm mesh to remove large particles and organisms, and then onto 3-µm polycarbonate membranes (142-mm diameter; Millipore, Billerica, MA, USA) to collect free-living Symbiodiniaceae cells. For comparison purposes, water samples were also collected from two locations devoid of corals; INC station was located inside and ONC station was located outside the Yongle Atoll, both far from coral presence. Samples from the INC station were collected near the bottom at a location where the water depth was 30 m, whereas samples from the ONC station were sampled 50 m below the surface at a location with a water depth of several hundred meters. The seawater samples collected from INC and ONC were filtered as described above.

Onboard, both the coral samples and seawater samples on filters were separately placed into a 2-mL tube containing 1-mL DNA lysis buffer (0.1 M EDTA and 1% SDS) and were preserved in liquid nitrogen. After arriving at the laboratory, these samples were stored at −80°C until DNA extraction was conducted.

### Environmental data collection

The monthly mean sea surface temperature (SST) for each sampling station was obtained from the US National Oceanic and Atmospheric Administration SST database with a temporal resolution of 1 d and a spatial resolution of approximately 4 km (51). The water depth was measured using an echo sounder. Salinity and pH were measured using a salinometer and pH meter, respectively. Water samples filtered through 0.22-µm polycarbonate membranes were used to measure the dissolved nutrient forms. Nitrate (NO_3_^-^), nitrite (NO_2_^-^), ammonium (NH_4_^+^), and phosphate (PO_4_^3-^) were analyzed by zinc-cadmium reduction, naphthalene ethylenediamine spectrophotometry, indophenol blue spectrophotometry, and phosphomolybdenum blue spectrophotometry, respectively. Dissolved inorganic nitrogen (DIN) was summed from NO_3_^-^, NO_2_^-^, and NH_4_^+^. Chlorophyll *a* (Chl *a*) was extracted in 90% acetone, and its concentration was measured as previously described (52). Detailed information on environmental factors is provided in Fig. S1.

### DNA extraction, PCR, and amplicon sequencing

The coral fragments preserved in the DNA lysis buffer were thawed at room temperature and then mashed in the DNA lysis buffer using a mortar and a pestle. DNA was extracted from a mixture of mashed coral powder and the DNA lysis buffer previously used to preserve the coral. Cell homogenization was conducted using an optimized bead beating protocol on an MP Cell Disruptor (MP Biomedicals, Irvine, CA, USA), and DNA was isolated using the cetyltrimethylammonium bromide (CTAB)-Zymo column method, as previously reported (53). The ambient free-living Symbiodiniaceae and other plankton preserved on the filter were thawed at room temperature. Subsequently, total DNA extraction was conducted as previously reported (54). DNA quantity and quality were measured using a NanoDrop 2000 spectrophotometer (Thermo Fisher Scientific, Franklin, MA, USA).

Twenty nanograms of the DNA obtained from each coral or ambient plankton sample was used as the template for polymerase chain reaction (PCR) to amplify the ITS2 region of Symbiodiniaceae rDNA. The 50-µL PCR mixture was prepared with 5 µL of TransStart buffer, 4 µL of dNTPs, 1 µL of each primer, 0.5 µL of TransStart Taq DNA polymerase, 38.5 µL distilled water, and the 20 ng template DNA. Primer sequences were ITS-DINO (5′-GTGAATTGCAGAACTCCGTG-3′) (9) and ITS2Rev2 reverse (5′-CCTCCGCTTACTTATATGCTT-3′) (55). The PCR program consisted of an initial denaturation at 95°C for 2 min, followed by 24 cycles of denaturation at 95°C for 45 s, annealing at 56°C for 45 s and elongation at 72°C for 45 s, with a final elongation step at 72°C for 5 min. The PCR products were purified, barcoded, pooled, and sequenced on the Illumina MiSeq platform using a 2 × 250 bp paired-end protocol from Azenta (Suzhou, China). The resulting raw sequences were deposited in the Sequence Read Archive of the NCBI BioProject (number PRJNA940648).

### Data processing and bioinformatics analysis

Because Symbiodiniaceae and other dinoflagellates rRNA genes have high intragenomic variations (56), amplicon sequence variant (ASV) data have the potential to inflate the estimate of diversity. A solution to minimize the problem is to use SymPortal (57). This framework uses the coral as the constraint to group the intragenomic variants (DIVs) within an individual coral into ITS2-type profiles, which are operationally coherent taxon units (e.g., genus). This method has been increasingly used (58–61) and hence employed in this study.

The coral and water samples of demultiplexed forward and reverse fastq.gz files were submitted to the SymPortal analytical framework (https://symportal.org) for quality control, including the removal of artefacts and non-Symbiodiniaceae sequences. After quality control processing, taxonomic screening, and minimum entropy decomposition analysis, the SymPortal output consists of the count table of ITS2 sequences (equivalent to the ASV feature table in Qiime) and ITS2-type profiles, and nucleotide sequences of ITS2 sequences (online supplementary file 1). The SymPortal ITS2 sequences, equivalent to ASVs in Qiime, were retrieved from each sample of coral and water samples. For brevity in referring to these analysis results, we name the SymPortal ITS2 sequences _S_ASVs from hereon. Furthermore, while _S_ASV data sets were generated for both coral and ambient water samples, *in hospite* symbiont _S_ASVs were directly subjected to ITS2-type profile identification in SymPortal, and the _S_ASVs found to be qualified to form profiles are named DIVs. Each ITS2-type profile exclusively belongs to a single genus. For example, in a *Cladocopium* ITS2-type profile designated as “C3/C3b-C3a-C3cc”, C3, C3b, C3a, and C3cc represent four DIVs, with their abundances decreasing in the specified order: C3 or C3b > C3a > C3cc; in some samples, C3 is the most abundant, and in other samples, C3b is the most abundant, and in all samples, C3cc is the least abundant and C3a is the second least abundant.

For water samples, ITS2-type profile identification cannot be directly carried out by SymPortal due to lack of constraint of coral host and potential lack of conspicuous dominant taxa. An indirect method was used. Representative _S_ASVs in ITS2-type profiles from coral samples can be used as a “bait” to detect the presence of the profile in the water sample data set (21). A phylogenetic tree was generated from the _S_ASVs sequences by aligning the sequences in Molecular Evolutionary Genetics Analysis (MEGA), then masking ambiguous alignments and inferring a tree using the SeaView (version 4.7) (62).

### Quantification of community assembly processes

The assembly model of different Symbiodiniaceae habitats (from hereon, the habitats refer to *P. damicornis*, *G. fascicularis*, and seawater) was investigated using the neutral community model (NCM) (63) and the Infer Community Assembly Mechanisms by Phylogenetic-bin-based null model analysis (iCAMP) (64). The data were organized based on the _S_ASVs using the minpack.lm, HMisc (65, 66), and iCAMP (64) package in R (version 4.0.5).

NCM was employed to assess the potential contribution of stochastic processes to the assembly of different Symbiodiniaceae communities. The goodness-of-fit (R^2^) value represents the overall fit of the NCM based on the least squares method, and the higher the R^2^, the greater influence community assembly receives from the stochastic processes and the less influence from the deterministic processes. Here, the lower the individual immigration rate from the source community to the local community (*m*), the more limited the species dispersal is in the whole community. Moreover, 95% confidence intervals for all fitted statistics were calculated from 1,000 bootstrapping replicates (65, 66).

The iCAMP framework quantifies the relative importance of different ecological processes based on their phylogenetic relationships between different groups (“bins”). These processes include dispersal limitation (DL), drift (DR), homogenizing dispersal (HD), heterogeneous selection (HeS), and homogeneous selection (HoS). Using this approach improves quantitative performance relative to the community-based approach (64). Fractions of DL, HD, and DR are largely considered stochastic, and summing their estimated relative importance can be used to estimate the stochasticity of community assembly (67).

To shed light on the underlying driving processes of the community structure, the modified stochasticity ratio (MST) was employed to quantitatively assess the community assembly process using the NST package in R (68): MST > 0.5, stochastic processes dominate; MST < 0.5, deterministic processes dominate. Differences among habitats were tested using the Kruskal-Wallis test in R.

### Statistical analysis

Statistical analyses of Symbiodiniaceae diversity were based on _S_ASVs data and conducted in the R and IBM SPSS statistics (version 20). The observed _S_ASV richness, Shannon-Wiener, Simpson, and phylogenetic diversity indices were calculated in R to assess the alpha diversity of _S_ASVs associated with coral and seawater samples. The differences in these four alpha diversity indices between coral and seawater samples were tested with non-parametric test (Kruskal-Wallis). To present the relationships between Symbiodiniaceae compositions in different samples from different habitats, a Bray-Curtis dissimilarity-based non-metric multidimensional scaling (NMDS) ordination analysis of Symbiodiniaceae compositions was performed using the vegan package in R (69). Permutational multivariate analysis of variance (PERMANOVA) and post-hoc pairwise test were carried out to examine differences in the Symbiodiniaceae composition. PERMANOVA was performed on Bray-Curtis distances with variance partitioned across site (H1, H2, H3, YY, JQ, HG, INC, and ONC), habitat (*P. damicornis*, *G. fascicularis*, and seawater), and geomorphic zone (outer reef slope, reef flat, inner reef slope, inner Yongle Atoll, and outer Yongle Atoll) defined in the model in this order.

Based on the similarity of latitudes and sea surface temperatures, the sampling stations in Hainan Island and Xisha Archipelago were divided into eight sites, from north to south, going from H1, H2, H3, YY, ONC, JQ, INC, and HG (Fig. 1). H1 included four stations: TGL (Tongguling), DJY (Dongjiao Yelin), CPG (Changpigang), and YL (Yinglong Bay); H2 consisted of two stations: JJ (Jiajing Island) and FJZ (Fenjiezhou Island); H3 comprised two stations: HH (Houhai) and LHT (Luhuitou). YY stands for Yinyu Island, JQ represents Jinqing Island, HG stands for Huaguang Reef, INC refers to inner Yongle atoll, and ONC refers to outer Yongle atoll (Table S1). The relative abundance of dominant symbiodiniacean taxa at different taxonomic levels was plotted using the ggplot2 package in R. The few missing values of environmental parameters were predicted by using the MICE package (70). Mantel test and variance partitioning analysis (VPA) were performed to evaluate the linkages between Symbiodiniaceae community structures of different habitats and environmental parameters, which were conducted using the vegan package.

## RESULTS

### Symbiodiniaceae community at the genus (clade) level

ITS2 sequencing followed by data quality-control processing, taxonomic screening, and minimum entropy decomposition analysis on the SymPortal framework yielded 7,568,023 high-quality Symbiodiniaceae sequencing reads across 114 coral samples and 69 seawater samples. The subsequent SymPortal analysis on the data set generated 835 _S_ASVs (Table S2), comprising the eight genera or genera-equivalent clades (3). *Cladocopium* (549 _S_ASVs) and *Durusdinium* (233) were dominant, and *Symbiodinium* (42), *Breviolum* (5), *Fugacium* (3), Clade I (2), *Freudenthalidium* (1), and *Halluxium* (1) were one or two orders of magnitude lower. The highest number of _S_ASVs was identified in seawater samples (465), followed by 288 _S_ASVs in *G. fascicularis* and 272 _S_ASVs in *P. damicornis*.

**Fig 2:**
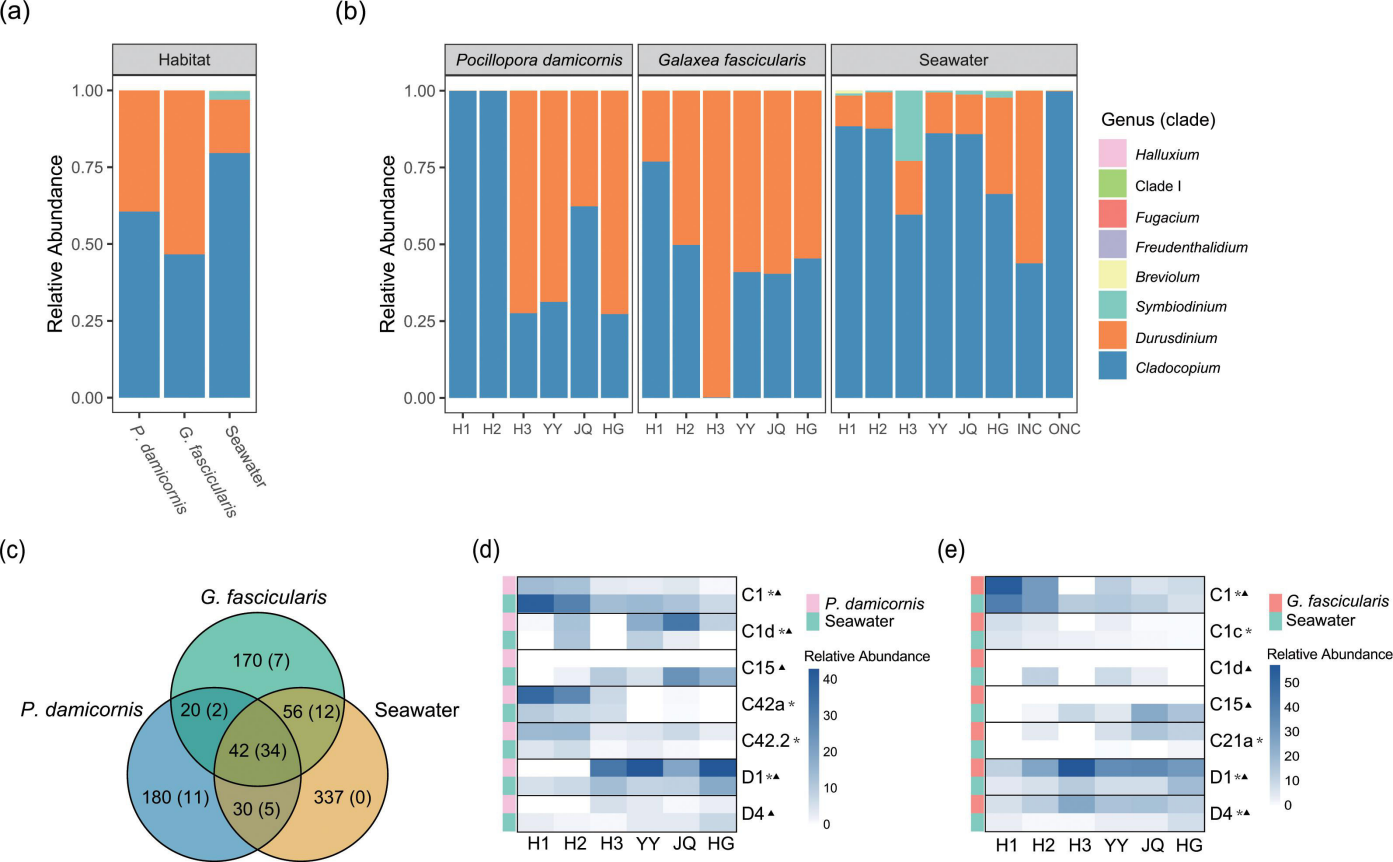
Symbiodiniaceae community profiles in different habitats and sites. Relative abundances of Symbiodiniaceae genera/clades in different habitats (**a**) and sites (**b**). (**c**) Venn diagram showing the overlaps of SymPortal Symbiodiniaceae ITS2 sequences (_S_ASVs) in different habitats, and the number of DIVs is in parentheses. (**d**) Comparison of the relative abundance of the top five dominant _S_ASVs in *P. damicornis* (asterisks) and the top five dominant _S_ASVs in seawater (triangle). (**e**) Comparison of the relative abundance of the top five dominant _S_ASVs in *G. fascicularis* (asterisks) and the top five dominant _S_ASVs in seawater (triangle).

*P. damicornis, G. fascicularis*, and seawater were characterized with significantly different Symbiodiniaceae compositions (Fig. 2a). *P. damicornis* contained a detectable but negligible level of *Symbiodinium* but overwhelmingly dominated by *Cladocopium* (58.08%) and *Durusdinium* (41.92%). Similarly, *Cladocopium* (42.30%) and *Durusdinium* (57.70%) predominated the symbiodiniacean community in *G. fascicularis*. In sharp contrast, the ambient water column had a much greater number of Symbiodiniaceae genera and genotypes. Eight genera were identified from the water column, with *Cladocopium* (77.24%) being the most abundant, followed by *Durusdinium* (19.14%), *Symbiodinium* (3.49%), and the least abundant taxa from *Breviolum*, *Fugacium*, *Freudenthalidium*, *Halluxium*, and Clade I. Despite the differences, *Cladocopium* and *Durusdinium* were consistently predominant in all our samples, and the contribution of *Cladocopium* was even higher in the water column than in corals.

Our data also showed a north-to-south gradient. Our study sites geographically ranged from 19.628239°N to 16.260556°N, and the *in hospite* Symbiodiniaceae community shifted from being dominated by *Cladocopium* in the northern area to being co-dominated by *Cladocopium* and *Durusdinium* in the southern area (Fig. 2b). Furthermore, differences were also observed between these two coral species. For *P. damicornis*, *Cladocopium* was predominant (i.e., accounting for >50% of reads in each sample) at sites H1, H2, and JQ, whereas *Durusdinium* was dominant at sites H3, YY, and HG. In *G. fascicularis*, in contrast, *Durusdinium* was dominant at all sites except for site H1, situated at the northern end of our study area, where the endosymbiont community was dominated by *Cladocopium*. In the ambient seawater samples, *Cladocopium* was dominant at all sites. Besides, the abundance of *Symbiodinium* in site H3 water was higher than at other sites, accounting for 22.84% of the free-living symbiodiniacean community. Between the water samples collected from the two no-coral sites, INC showed a similar symbiodiniacean community composition to coral-ambient sites, with *Cladocopium* and *Durusdinium* being the major contributors, whereas ONC was dominated by *Cladocopium* (99.79%).

### Symbiodiniaceae ITS2-type profiles

From our 114 coral samples, 71 DIVs (Fig. S2) were identified, which produced 62 Symbiodiniaceae ITS2-type profiles (Fig. S3; Table S3). Thirty-seven of these profiles belonged to the genus *Cladocopium*, and the remaining 25 to *Durusdinium*. Similar to _S_ASV data shown above, the ITS2-type profile also showed greater dominance of *Cladocopium* in the corals.

Of the 62 total coral-associated ITS2-type profiles, 30 occurred in *P. damicornis*, 19 of which were *Cladocopium* with the remaining 11 being *Durusdinium*. Whereas, *P. damicornis* from northern sites, H1 and H2, harbored *Cladocopium* profiles, those from the southern sites, H3, YY, JQ, and HG, hosted both *Cladocopium* and *Durusdinium* profiles. The major *Cladocopium* profiles included C42a/C1/C42.2-C42b-C1az-C1b-C1au-C115d and C1d-C42.2-C1-C1k-C1b-C3cg, and major *Durusdinium* profiles were D1-D2d-D6-D4-D2.2-D4t-D2f and D1-D1ju-D2d-D4t (Fig. S3).

Thirty-three ITS2-type profiles were identified across all *G. fascicularis* samples, 19 of which were *Cladocopium* with the remaining 14 being *Durusdinium. G. fascicularis* harbored both *Cladocopium* and *Durusdinium* profiles from all study sites, including the northern sites H1 and H2, whereas *P. damicornis* harbored only *Cladocopium,* as shown above. Therefore, sites H1 and H2 seemed to mark a shift of environmental conditions (likely temperature) that physiologically set the two coral species apart. The major *G. fascicularis*-associated *Cladocopium* profiles were C21a-C21-C3-C21k, C1/C3-C1c-C1b-C1w, and C1/C1c-C1b-C72k-C42.2, and *Durusdinium* profiles were mainly D1/D4-D4c-D2-D1h, D1-D4-D4c-D1c-D2, and D1-D4-D4c-D4f-D3b (Fig. S3). The genotype composition in each profile was also somewhat different between the two coral species.

Complete sets of *Cladocopium* and *Durusdinium* DIV sequences (as defined by SymPortal ITS2-type profiles) were retrieved from 68 out of the 69 water samples in total (Table S4). Out of the 30 ITS2-type profiles identified in *P. damicornis* described above, 16 were recovered from 66 water samples. The top three most abundant profiles were D4, C15/C1, and C3u/C3, with their full sets of DIVs recovered from 56, 46, and 31 water samples, respectively. The major *Cladocopium* profile C1d-C42.2-C1-C1k-C1b-C3cg, found in four *P. damicornis* samples, occurred in three water samples, with its full set of DIVs being retrieved. Of the 33 ITS2-type profiles identified in *G. fascicularis* samples (described above), 25 were recovered from 66 water samples. The top three most abundant profiles were C1, C3u, and D1/D4/D4c, with their full sets of DIVs being recovered from 63, 32, and 28 water samples, respectively. All DIVs of the six major *G. fascicularis*-associated *Cladocopium* and *Durusdinium* profiles were all retrieved from the water samples. These data considered at the ITS2-type profile level indicated substantial overlap between the *in hospite* and the ambient *ex hospite* symbiodiniacean assemblages.

### Symbiodiniaceae community at _S_ASV level

*P. damicornis* and *G. fascicularis* shared 72 and 98 _S_ASVs (39 and 46 of which belonged to DIVs, respectively), respectively, with the water column (Fig. 2c). All three habitats (*P. damicornis*, *G. fascicularis*, and seawater) shared 42 _S_ASVs (34 of which belonged to DIVs) (Fig. 2c), and these _S_ASVs comprised >50% of the Symbiodiniaceae assemblages (Fig. S4). Furthermore, the top five dominant _S_ASVs of the two coral species and water column were included in these 34 DIVs. The D1 (23.07%), C42a (12.36%), C1d (12.30%), C42.2 (7.85%), and C1 (6.25%) were top five dominant _S_ASVs in *P. damicornis*, whereas in *G. fascicularis*, the D1, C1, D4, C21a, and C1c were the most abundant, accounting for 33.14%, 18.95%, 15.27%, 6.5%, and 3.01%, respectively. In the water column, the C15, C1, D1, D4, and C1d contributed the most to _S_ASVs of the community (19.19%, 15.14%, 12.23%, 4.18%, and 4.06%, respectively). Upon comparison, we found that some dominant _S_ASVs of each coral overlap with those in the ambient water column. Additionally, we observed that the top five dominant _S_ASVs of *P. damicornis* coexisted in the water column (Fig. 2d), while the majority of the top five dominant _S_ASVs of *G. fascicularis* also coexisted in the water column (Fig. 2e). Therefore, each coral species appears to harbor some symbiodiniacean taxa that exist in the ambient environment. However, C15, the dominant _S_ASV in water samples, was rarely present in the two species of corals.

### The alpha and beta diversity of Symbiodiniaceae communities

**Fig 3:**
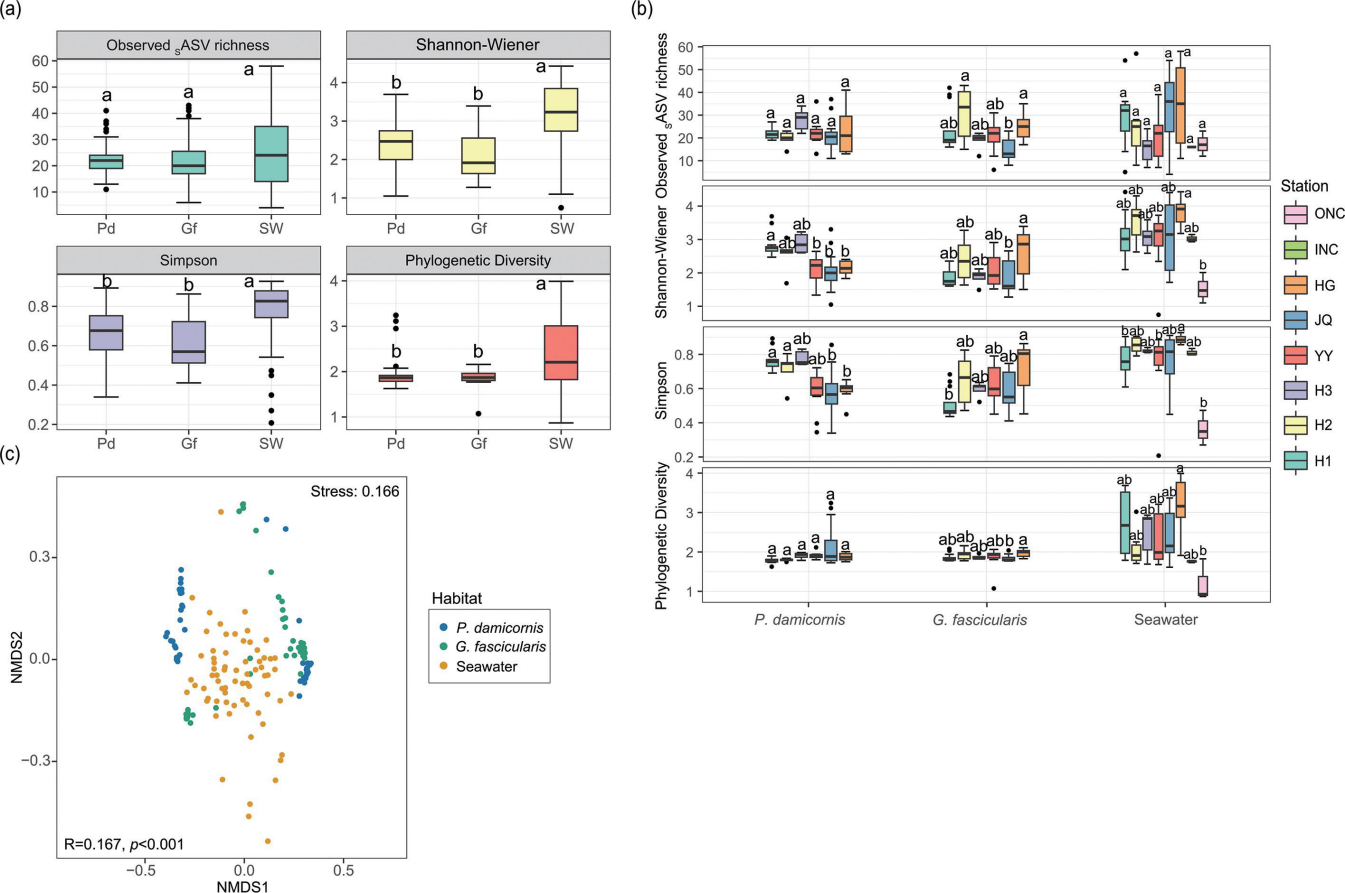
Changes in Symbiodiniaceae alpha diversity in different habitats (**a**) and sites (**b**). The boxplots are constructed with the first and third quartiles of the distribution of values and medians. The lines extending from the boxes indicate the variability outside the first and third quartiles. Data are expressed as mean ± SD. Significant differences (*P* < 0.05) are indicated by different letters. Pd, *Pocillopora damicornis*; Gf, *Galaxea fascicularis*; SW, seawater. (**c**) Two-dimensional NMDS ordination using Bray–Curtis indices of Symbiodiniaceae communities in different habitats. Analysis of similarities (ANOSIM) of the effect of habitat on Symbiodiniaceae community similarity. R value and *P* value are shown in the lower left corner.

Four alpha diversity indices were estimated based on _S_ASV data to estimate the alpha diversity of Symbiodiniaceae communities in the different habitats and sites (Fig. 3a and b). The results showed that the three alpha diversity indices changed significantly in the different habitats (Fig. 3a). The observed _S_ASV richness of *P. damicornis* and *G. fascicularis* was lower than that of the water column, although this difference was not statistically significant. The Shannon-Wiener, Simpson, and phylogenetic diversity index values were all significantly higher in the water column than in the corals (*P. damicornis* and *G. fascicularis*) (*P* < 0.05). The observed _S_ASV richness, Shannon-Wiener, and Simpson indices of the two coral species were lower in *G. fascicularis* than in *P. damicornis* (albeit without statistical significance).

The alpha diversity at different sites in the same habitat also varied significantly (Fig. 3b). In *P. damicornis*, the Shannon-Wiener and Simpson index values in Hainan Island (H1, H2, and H3) were higher than those in the Xisha Archipelago (YY, JQ, and HG). In *G. fascicularis*, there was no remarkable difference between Hainan Island and Xisha Archipelago. In the water column, the four alpha diversity indices in INC were not significantly different from those of the coral-surrounding seawater. Notably, at ONC, there was no significant difference in the observed _S_ASV richness with other sites, but Shannon-Wiener, Simpson, and phylogenetic diversity index values were lower than other sites.

NMDS ordination revealed that the Symbiodiniaceae communities were grouped by habitat (*P. damicornis*, *G. fascicularis*, and water column) (analysis of similarities [ANOSIM] R-value: 0.167; *P* < 0.001) (Fig. 3c). PERMANOVA analyses (Table 1; Table S5) indicated that site had the greatest impact on Symbiodiniaceae community structure (R^2^ = 0.193; *P* < 0.001), followed by habitat (R^2^ = 0.157; *P* < 0.001), and geomorphic zone (R^2^ = 0.030; *P* < 0.05). Of the two-factor interactions, site × habitat (R^2^ = 0.104; *P* < 0.001), site × geomorphic zone (R^2^ = 0.027; *P* < 0.001), and habitat × geomorphic zone (R^2^ = 0.045; *P* < 0.001) were significant. Of the three-factor interactions, site × habitat × geomorphic zone (R^2^ = 0.040; *P* < 0.001) was significant. The results of the post-hoc pairwise test are provided in Table S5.

**TABLE 1:** Summary of PERMANOVA output, with the partitioning of variance in a Bray-Curtis distance matrix of SymPortal Symbiodiniaceae ITS2 sequences (_S_ASVs) abundances^*a*^

Factor	F.model	R^2^	Pr (>F)
Site	10.051	0.193	0.001
Habitat	28.609	0.157	0.001
Geomorphic_zone	5.437	0.03	0.001
Site:Habitat	3.792	0.104	0.001
Site:Geomorphic_zone	2.492	0.027	0.001
Habitat:Geomorphic_zone	4.118	0.045	0.001
Site:Habitat:Geomorphic_zone	2.91	0.04	0.001
Residuals		0.405	

^*a*^ The model was specified with site (eight levels), habitat (three levels), and geomorphic zone (five levels) as factors. Site: H1, H2, H3, YY, HG, JQ, INC, and ONC. Habitat: *in hospite*/*Pocillopora damicornis*, *in hospite*/*Galaxea fascicularis*, and free living/seawater. Geomorphic zone: outer reef slope, reef flat, inner reef slope, inner Yongle Atoll, and outer Yongle Atoll.

### Symbiodiniaceae compositions in relation to environmental variables

**Fig 4:**
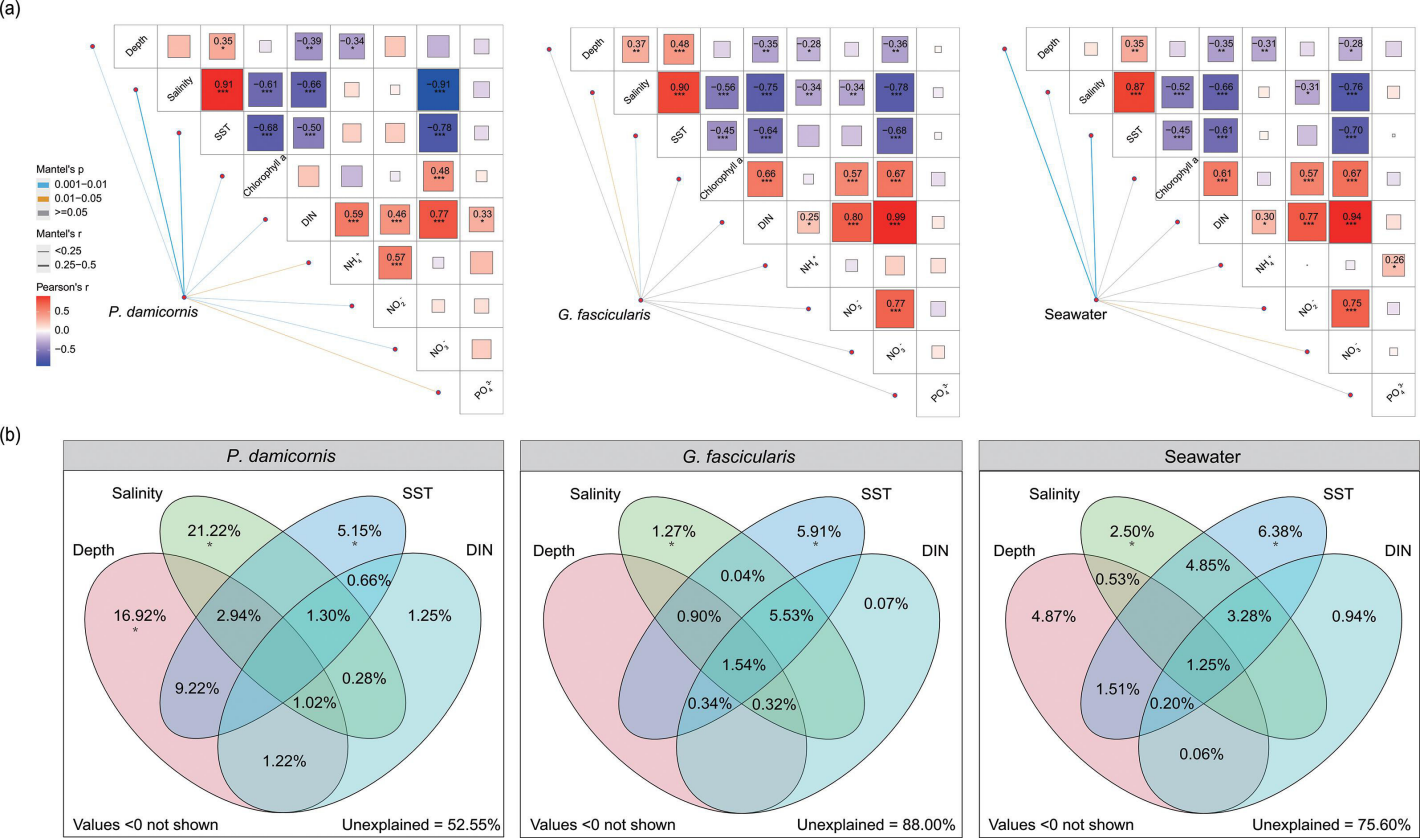
(**a**) Environmental drivers of Symbiodiniaceae community in different habitats evaluated by Mantel test. Edge width and color correspond to R value and *P* value, respectively. Color gradients represent Spearman correlation coefficients between environmental variables. Asterisks indicate significance (*** *P* < 0.001; ** *P* < 0.01; * *P* < 0.05). (**b**) Venn diagrams of variance partitioning analysis showing the effects of environmental factors on the Symbiodiniaceae communities. The percentage of variation explained by each factor, including unique, shared, and unexplained, is shown in corresponding positions in the diagram. When no numbers are shown, this matrix had no explanatory wer (zero or negative). Asterisks represent statistical significance: *P < 0.05, as estimated using canonical correspondence analysis.

The environmental factors used in the analysis were depth, salinity, SST, Chl *a*, DIN, NH_4_^+^, NO_2_^-^, NO_3_^-^, and PO_4_^3-^. Their correlations with Symbiodiniaceae community composition in different habitats were analyzed using the Mantel test (Fig. 4a; Table S6). Symbiodiniaceae communities in *P. damicornis* exhibited significant positive correlations with all environmental factors. Symbiodiniaceae communities in *G. fascicularis* were positively correlated only with salinity and SST. Moreover, Symbiodiniaceae communities in the water column showed a significant positive correlation with depth, salinity, SST, and NO_3_^-^.

VPA was performed to quantify the relative contribution of environmental parameters on the Symbiodiniaceae community. Based on VPA (Fig. 4b), salinity, depth, and SST were identified as the most important environmental factors shaping the Symbiodiniaceae community in *P. damicornis*, with conditional effects of 21.22%, 16.92%, and 5.15%, respectively (*P* < 0.05). Moreover, in *G. fascicularis*, SST and salinity were identified as the most important environmental factors shaping the Symbiodiniaceae community, with conditional effects of 5.91% and 1.27%, respectively (*P* < 0.05). Similarly, SST and salinity were also identified as the most important environmental factors shaping the Symbiodiniaceae community in the water column, with conditional effects of 6.38% and 2.50%, respectively (*P* < 0.05). Notably, both depth and SST had significant effects on the Symbiodiniaceae communities of the three different habitats.

### Ecological assembly processes of Symbiodiniaceae communities

**Fig 5:**
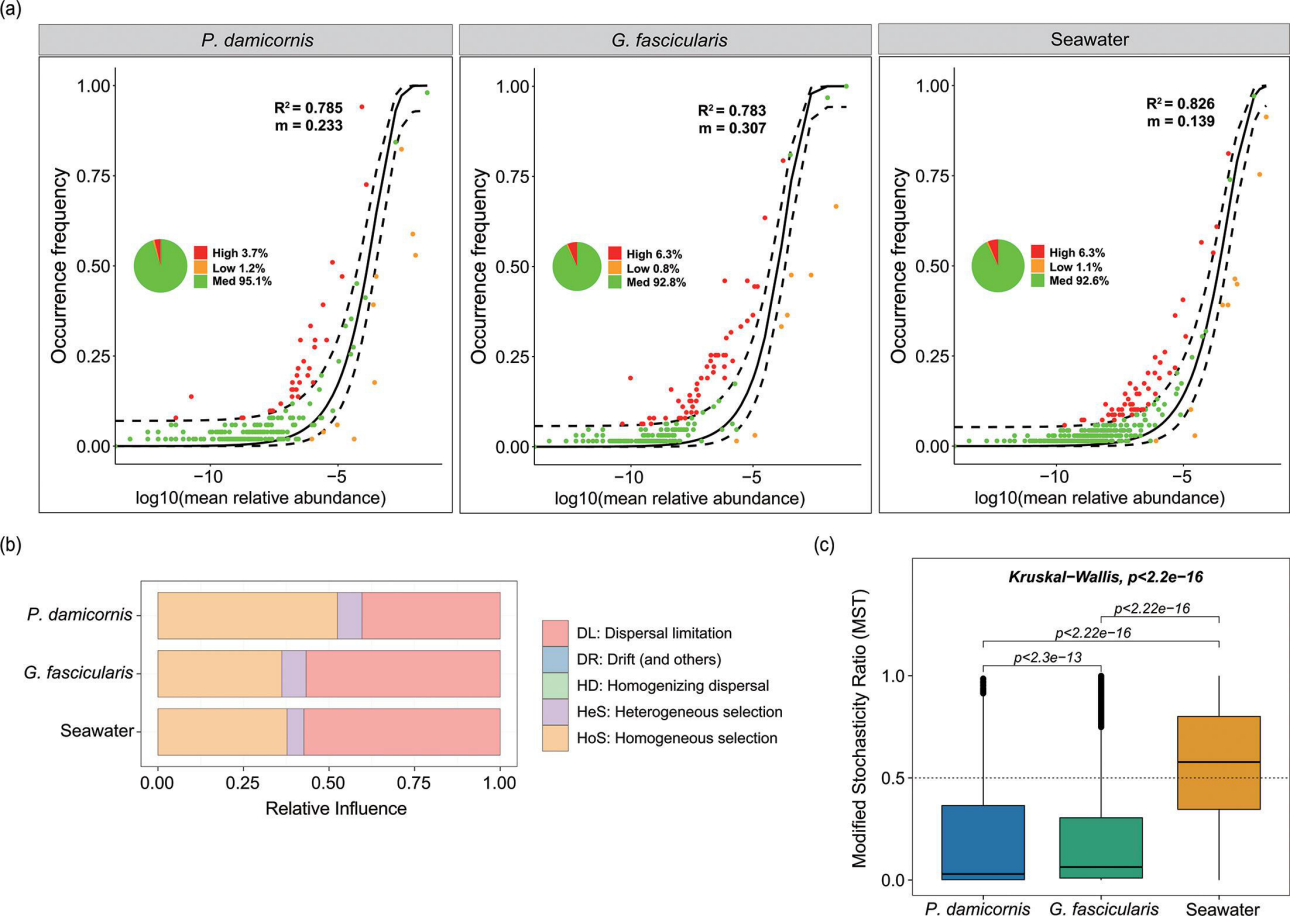
(**a**) Implementation of a neutral model for analysis of Symbiodiniaceae community structure in different habitats. Dashed lines represent 95% confidence intervals around the best-fit neutral model. The _S_ASVs occurring more and less frequently than that predicted by the model are shown in red and orange points, respectively; the green points are within the confidence intervals. (**b**) The relative importance of different ecological processes in the assembly of Symbiodiniaceae communities with different habitats based on iCAMP analysis. (**c**) Modified stochasticity ratio indicating quantified Symbiodiniaceae community assembly processes. MST > 0.5, stochastic processes dominate; MST < 0.5, deterministic processes dominate.

The neutral community model (NCM) was used to investigate the contribution of stochastic processes of Symbiodiniaceae community assembly (Fig. 5a). Symbiodiniaceae compositions fit a neutral model when the three habitats were individually considered. The R^2^ values were 0.785 for *P. damicornis*, 0.783 for *G. fascicularis*, and 0.826 for seawater as source communities. The neutral interpretation provided a better fit for seawater communities than coral communities. The majority of the _S_ASVs fell within the 95% confidence intervals of the models, with values from 92.6% to 95.1%, indicating that the models predicted well. Concurrently, the immigration rate *m* was 0.233 for *P. damicornis*, 0.307 for *G. fascicularis*, and 0.139 for seawater. The higher *m* values of *P. damicornis* and *G. fascicularis* than seawater signaled that the impact of dispersal limitation on the free-living community was stronger than that on the coral-endosymbiotic Symbiodiniaceae communities.

Based on the iCAMP analysis (Fig. 5b; Table S7), the observed 835 _S_ASVs were divided into 18 phylogenetic bins. Homogeneous selection (52.44%) was the most decisive factor in determining the assembly process of Symbiodiniaceae communities in *P. damicornis*, followed by DL (40.38%) and heterogeneous selection (7.18%). For *G. fascicularis*, DL (56.70%) and HoS (36.20%) were the predominant ecological processes, although HeS (7.09%) was also important. In the water column, DL (57.35%) and HoS (37.73%) contributed more to community assembly, whereas HeS (4.92%) had a low estimated relative importance. The results showed the relative importance of stochastic processes in the seawater column was higher than in *G. fascicularis* and *P. damicornis*, with stochastic processes accounting for 57.35%, 56.71%, and 40.38% of the assembly mechanism (Table S8). Overall, the Symbiodiniaceae community was more strongly shaped by HoS and DL than by HeS and others in both the corals and the water column. Therefore, the assembly of both free-living and coral-endosymbiotic Symbiodiniaceae communities was governed by both deterministic and stochastic processes.

The MST analysis results showed that the MST values for *P. damicornis* and *G. fascicularis* were both less than 0.5, while that for seawater was greater than 0.5 (Fig. 5c). These indicated that deterministic processes played a leading role in the assembly of *in hospite* Symbiodiniaceae communities, while stochastic processes played a decisive role in the community assembly of ambient environmental Symbiodiniaceae. Additionally, the Kruskal-Wallis test showed significant differences among the three habitats.

## DISCUSSION

### Ambient Symbiodiniaceae community has higher diversity than *in hospite* assemblage but with shared dominant genotypes

To address our first hypothesis that ambient Symbiodiniaceae community has higher richness and diversity than, but with the same dominant genotypes as, *in hospite* assemblage, ITS2-based taxon richness and diversity were compared between *in hospite* and ambient communities of Symbiodiniaceae. From our samples, eight genera/clades of Symbiodiniaceae (*Cladocopium*, *Durusdinium*, *Symbiodinium*, *Breviolum*, *Fugacium*, *Freudenthalidium*, *Halluxium*, and Clade I) were retrieved from the water column, which is generally consistent with the results of previous studies on corals (19, 45, 46, 71, 72). The only exception is *Gerakladium*, which was previously reported from the water column in Sanya (19), but not detected in our study. In addition, our detection of clade I population in one of our water samples, albeit at a low abundance, is interesting because it has only been reported in corals (in low abundances) in the SCS (46, 73). These small discrepancies might reflect the temporal variations or inadequate sequencing of the environmental Symbiodinaceae communities in previous studies. Of the eight symbiodiniacean genera found in the water column, only three genera (*Cladocopium*, *Durusdinium*, and *Symbiodinium*) were detected in the two coral species we investigated, *P. damicornis* and *G. fascicularis*. However, the other four genera have previously been found in corals in the SCS but in other coral species. For instance, previous studies have shown *Breviolum* and clade I in the coral species *Acropora valida*, *Acropora formosa*, *Dipsastraea favus*, and *Plesiastrea versipora* (46, 72), and *Halluxium* in coral species *Goniastrea aspera* (73). Corals *Porites lutea* and *Favia palauensis* were found to contain the genera *Gerakladium* and *Fugacium* (46). Furthermore, corals *Montipora efflorescens*, *Acropora valida*, and *Turbinaria peltata* were also found to contain the same three genera (*Cladocopium*, *Durusdinium*, and *Symbiodinium*) of Symbiodiniaceae found in *P. damicornis* and *G. fascicularis* (46, 72). Based on data from the present and previous studies (45, 46, 71, 73–76), except for *Freudenthalidium*, which only appears in the water column, eight genera/clades of Symbiodiniaceae (*Cladocopium*, *Durusdinium*, *Symbiodinium*, *Breviolum*, *Fugacium*, *Gerakladium*, *Halluxium*, and Clade I) occur in both the water column and coral community in the SCS, but with different combinations of genera/clades living symbiotically within different coral species. Notably, although *Durusdinium* was the dominant genus in both *P. damicornis* and *G. fascicularis* at certain sites, *Cladocopium* was invariably dominant in the water column. Differences between *in hospite* and free-living Symbiodiniaceae communities at the genus level suggest selective symbiont recruitment (77).

The four alpha diversity indices were also higher in the water column than in individual coral species (*P. damicornis* and *G. fascicularis*), indicating that regardless of coral type, richness and diversity were higher in free-living Symbiodiniaceae communities than in coral-endosymbiotic Symbiodiniaceae communities. This agrees with the results discussed above based on the number of genus/clade and _S_ASVs, and may, in part, reflect that not all free-living Symbiodiniaceae can form symbiotic relationships (31, 77, 78). The three alpha diversity indices (observed _S_ASV richness, Shannon-Wiener, and Simpson) were higher in *P. damicornis* than in *G. fascicularis*, indicating that richness and diversity were higher in the Symbiodiniaceae communities of a sensitive coral than that of a bleaching-resistant coral (45, 49). In *P. damicornis*, the two alpha diversity indices (Shannon-Wiener and Simpson) in Hainan Island were higher than those in the Xisha Archipelago, particularly in H3 (Fig. 3b). This suggests a stronger selection pressure in the southern (warmer) area. In the water column, no significant differences were observed among the six sites (H1, H2, H3, YY, JQ, and HG) where coral samples and their corresponding ambient water samples were collected simultaneously. Interestingly, at the no-coral site INC, the alpha diversity indices resembled those of the six sites (H1, H2, H3, YY, JQ, and HG) around coral reefs, although the indices at the no-coral site ONC appeared to be lower than those of the six sites (but without statistical significance). The differences in alpha diversity between INC and ONC were similar to the variations in symbiodiniacean taxa composition.

The ITS2-type profile results indicate that both *P. damicornis* and *G. fascicularis* are highly flexible in symbiont associations, but there are marked differences between them (Fig. S3). From our data set, 62 Symbiodiniaceae ITS2-type profiles were predicted from *P. damicornis* and *G. fascicularis* samples, 41 of which had complete sets of DIV sequences retrieved in almost all water samples. Additionally, there was an overlap in the dominant _S_ASVs for each coral and ambient water column in different sites, especially D1 and C1 (Fig. 2d and e). Therefore, these results revealed a high degree of taxon overlap between free-living and *in hospite* symbiodiniacean communities, and these overlaps contain dominant genotypes in both the *in hospite* and ambient habitats. Besides, the taxon richness is higher in the ambient environment than within individual corals, although collectively, all *in hospite* Symbiodiniaceae taxa combined largely match that in the ambient water column. Hence, our data verify hypothesis 1 that ambient Symbiodiniaceae community has higher diversity than *in hospite* assemblage but with shared dominant genotypes.

In general, Symbiodiniaceae reservoirs allow corals to reshuffle their symbiont composition in response to environmental stress to achieve resilience (21, 26, 30–32). For the SCS, the ambient free-living symbiodiniacean community is qualified as a source of symbionts for corals. The diverse gene pool we found in the ambient environment will enable corals with horizontal transmission to acquire symbionts from the environment in each offspring generation (25). Between the two coral species we studied here, *G. fascicularis* had more _S_ASVs overlapping with the water column than *P. damicornis*, suggesting that the exchange with environmental Symbiodiniaceae in the water column probably occurs more frequently for *G. fascicularis* than for *P. damicornis*. Furthermore, free-living Symbiodiniaceae also occur in sediments, and the sediment flora is also an important source for recruitment of symbionts by both horizontally transmitting adult corals and coral larvae (17, 79). Because sediment samples were not collected in our study, the relative importance of water column versus sediment Symbiodiniaceae cannot be assessed. Future research should include sampling of sediments, and the findings here provide a basis for formulating a testable hypothesis.

### Similar environmental and geographical effects on free-living and *in hospite* Symbiodiniaceae communities

Our hypothesis 2 states that environmental factors have similar effects on *in hospite* and ambient Symbiodiniaceae communities. To address this hypothesis, we conducted Mantel test, VPA, and PERMANOVA analyses to explore the factors influencing Symbiodiniaceae communities under different lifestyles. Based on Mantel test results, Symbiodiniaceae communities in *P. damicornis* showed significant correlations with all environmental factors, whereas *G. fascicularis* was significantly correlated only with salinity and SST. These results are consistent with those of previous studies showing that *P. damicornis* is sensitive to environmental conditions, whereas *G. fascicularis* is a resistant species (49, 50). The reproductive modes of *P. damicornis* include both brooding and broadcast spawning (80), while *G. fascicularis* involves broadcast spawning (81). Compared to *G. fascicularis*, the top five dominant _S_ASVs of *P. damicornis* were observed in almost all adjacent water samples at each site (Fig. 2d). For *P. damicornis* as a susceptible species, acquiring a more flexible *in hospite* Symbiodiniaceae composition through a brooding plus broadcast spawning reproductive strategy might enable the species to better cope with environmental stress.

Nevertheless, in the water column, Symbiodiniaceae communities exhibited statistically significant correlations with depth, salinity, SST, and NO_3_^-^. Thus, it appears that ambient free-living Symbiodiniaceae is an intermediate between *P. damicornis* and *G. fascicularis* in terms of their susceptibility to environmental variations. Among *Cladocopium* types, type C15 and closely related variants have been shown to be more thermally tolerant, with greater photosynthetic stability in response to extreme environmental conditions (82, 83). In the present study, C15 and C15h in the water column were much more abundant than in corals, particularly at the ONC (50-m depth) (Fig. S4). It is generally believed that C1 is a thermosensitive type (84), whereas D1 and D4 are thermotolerant types (22, 85). C1d dominates the Symbiodiniaceae community in bleaching coral reefs in Pandora Reef, Australia (86). In our study, we observed that the abundances of C1 and C42a at Hainan Island (lower temperature) were higher than that at the Xisha Archipelago (higher temperature), while the abundances of C1d, C15, D1, and D4 were lower than that at the Xisha Archipelago (Fig. S4). Therefore, the northern area had more Symbiodiniaceae thermosensitive genotypes, whereas the southern area had more Symbiodiniaceae thermotolerant genotypes. Furthermore, the H3 site in Sanya appears to be an inflection point of the Symbiodiniceae community structure while geographically marking the transition from Hainan Island to the Xisha Archipelago. H3 had a higher abundance of *Durusdinium* than H1 and H2 on Hainan Island, and its symbiodiniacean community was more similar to the sites in the Xisha Archipelago, which may have been detrimentally affected by factors such as elevated temperature, increased anthropogenic disturbances, sedimentation, and freshwater incursion (19, 45, 71).

According to Mantel test and VPA results, the most important environmental factors and their contributions differ in different habitats, indicating that the driving factors of Symbiodiniaceae communities vary with the host species and the lifestyle of Symbiodiniaceae. Previous studies have shown that the Symbiodiniaceae communities in corals are mainly influenced by temperature and salinity (36, 37). Similarly, SST and salinity appeared to be important in shaping the free-living Symbiodiniaceae community. The PERMANOVA analyses showed that three factors (site, habitat, geomorphic zone) and their interactions had a significant impact on the Symbiodiniaceae community structure (*P* < 0.05), indicating that free-living Symbiodiniaceae communities, similar to *in hospite* Symbiodiniaceae communities, are also influenced by geographical location and geomorphic zone (7, 19, 30). Thus, our results verify hypothesis 2 that similar environmental and geographical effects impact *in hospite* and ambient Symbiodiniaceae communities.

### Different assembly mechanisms for the free-living and *in hospite* Symbiodiniaceae communities

The final hypothesis in the current study predicts that different assembly mechanisms dominate the assembly of *in hospite* and ambient free-living Symbiodiniaceae communities. To examine if this is consistent with our data, two different conceptual frameworks respectively based on neutral mode (63) and null model (64) were used to investigate the assembly processes of Symbiodiniaceae communities in different lifestyles. We found that the assembly of free-living and coral-endosymbiotic Symbiodiniaceae communities was governed by both deterministic and stochastic processes. Nevertheless, deterministic processes dominated *in hospite* Symbiodiniaceae community assembly, and the assembly of free-living Symbiodiniaceae communities was mainly determined by stochastic processes. Community assembly of phytoplankton and bacterioplankton (free-living and attached forms) is also often regulated by a combination of deterministic and stochastic processes, but as the environment changes, the dominant process will also change (38, 87, 88).

Stochastic processes have a greater influence on planktonic bacterial and archaeal community assembly than deterministic processes, attributed to the stronger adaptive capabilities of marine prokaryotes to environmental changes, and spatial connectivity and homogenizing effects of seawater movement on environmental conditions (89). Similarly, in the present study, the NCM analysis results revealed the importance of stochastic processes in shaping the composition and diversity of Symbiodiniaceae communities in free-living form was higher than that in coral-endosymbiont forms. The iCAMP analysis yielded similar results and showed that DL had a stronger effect on free-living Symbiodiniaceae communities than on coral-endosymbiont Symbiodiniaceae communities. Isabwe et al. (88) argue that despite the high passive dispersal abilities of plankton communities in lotic environments, there are limitations to the extent of their dispersal. Free-living Symbiodiniaceae are more susceptible to predation in the environment compared to endosymbiotic Symbiodiniaceae (90), whereas predators can increase the importance of stochastic processes by reducing the number of individuals surviving in each environment (91). Furthermore, stochastic processes cause divergence in community composition with similar environments (91). Dispersal limitation also leads to community divergence due to limited exchange of microbes (92). Although the abundance of free-living Symbiodiniaceae is orders of magnitude lower than that of symbiotic Symbiodiniaceae (90), they exhibited higher richness and diversity.

In addition, a novel finding of this study was that the deterministic processes dominated the coral-endosymbiotic Symbiodiniaceae community assembly in both bleaching-sensitive and bleaching-resistant corals (Fig. 5c). Abiotic environmental factors, such as temperature, oxygen, and nutrient concentrations, and biotic interactions, such as predation, competition, and mutualism, are likely responsible for the community differences (44, 71, 93, 94). The algal host can affect the bacterial community assembly on the microalgal phycosphere, and deterministic processes play an important role (95). Furthermore, the phytoplankton *Phaeocystis globosa* is selective for the recruitment of symbiotic microbiome, and deterministic processes dominate symbiotic microbiome community assembly (96). Therefore, mutualism may also play a decisive role in shaping the community structure of coral-endosymbiotic Symbiodiniaceae, regardless of coral type. Furthermore, deterministic processes (i.e., environmental filtering and species interaction) in coral-endosymbiont Symbiodiniaceae communities were dominated by HoS. We found a different relative importance of HoS in two different ecotypes of coral, with *P. damicornis* (52.44%) influenced by the greatest number of environmental factors and had higher alpha diversity, and *G. fascicularis* (36.20%) by the fewest factors and lower alpha diversity, evidencing that the Symbiodiniaceae community of *P. damicornis* was more significantly affected by environmental factors, while that of *G. fascicularis* was more strongly selected by host species. As a result, hypothesis 3 is also verified.

### Conclusions

In this study, we investigated the distribution, diversity, driving factors, and assembly mechanisms of free-living and *in hospite* Symbiodiniaceae communities in the northern South China Sea, utilizing ITS2 rRNA gene high-throughput sequencing. Our results verify all three hypotheses raised in this work. First, the data reveal that free-living Symbiodiniaceae community has higher richness and diversity than *in hospite* Symbiodiniaceae community in the two coral species we studied, with eight genera/clades detected in the ambient water and three in the corals. Second, we find that the environmental and geographical factors, dominantly SST, salinity, and geographical location, exert similar effects on *in hospite* and ambient Symbiodiniaceae communities. Third, our results indicate that the assembly of free-living and coral-endosymbiotic Symbiodiniaceae communities was governed by both deterministic and stochastic processes. However, deterministic processes (environmental filtering and species interaction) exhibited stronger effects on the symbiotic communities, whereas stochastic processes, particularly dispersal limitation, had stronger effects on the free-living community. As such, this study presents a new perspective and foundation for future works on the adaption and resilience of corals in response to environmental variations and anthropogenic disturbances.

## Data Availability

Raw sequence data are deposited in the SRA (BioProject PRJNA940648).

## SUPPLEMENTAL MATERIAL

Supplemental figures spectrum.00514-24-s0001.docx Fig. S1–S4.

Supplemental material spectrum.00514-24-s0002.txt The nucleotide sequences of 835 SymPortal Symbiodiniaceae ITS2 sequences (sASVs).

Supplemental tables spectrum.00514-24-s0003.xlsx Table S1–S8.

ASM does not own the copyrights to Supplemental Material that may be linked to, or accessed through, an article. The authors have granted ASM a non-exclusive, world-wide license to publish the Supplemental Material files. Please contact the corresponding author directly for reuse.
