# Supplementary material for: Higher genotypic diversity and distinct assembly mechanism of free-living Symbiodiniaceae assemblages than sympatric coral-endosymbiotic assemblages in a tropical coral reef
Source: Microbiol Spectr. 2024 Jun 14;12(8):e00514-24. doi: 10.1128/spectrum.00514-24 (PMC11302235; doi:10.1128/spectrum.00514-24)
Supplement: Supplemental figures — Fig. S1–S4. [file spectrum.00514-24-s0001.docx]

**Supplementary Figure**

Figure S1. An overview of environmental data at each site. The boxplots are constructed with the first and third quartiles of the distribution of values and the medians. The lines extending from the boxes indicate the variability outside the first and third quartiles. Data are expressed as mean ± sd.

Figure S2. The relative abundance of Symportal Symbiodiniaceae DIVs in different habitats at different sites (a) and in each sample (b).


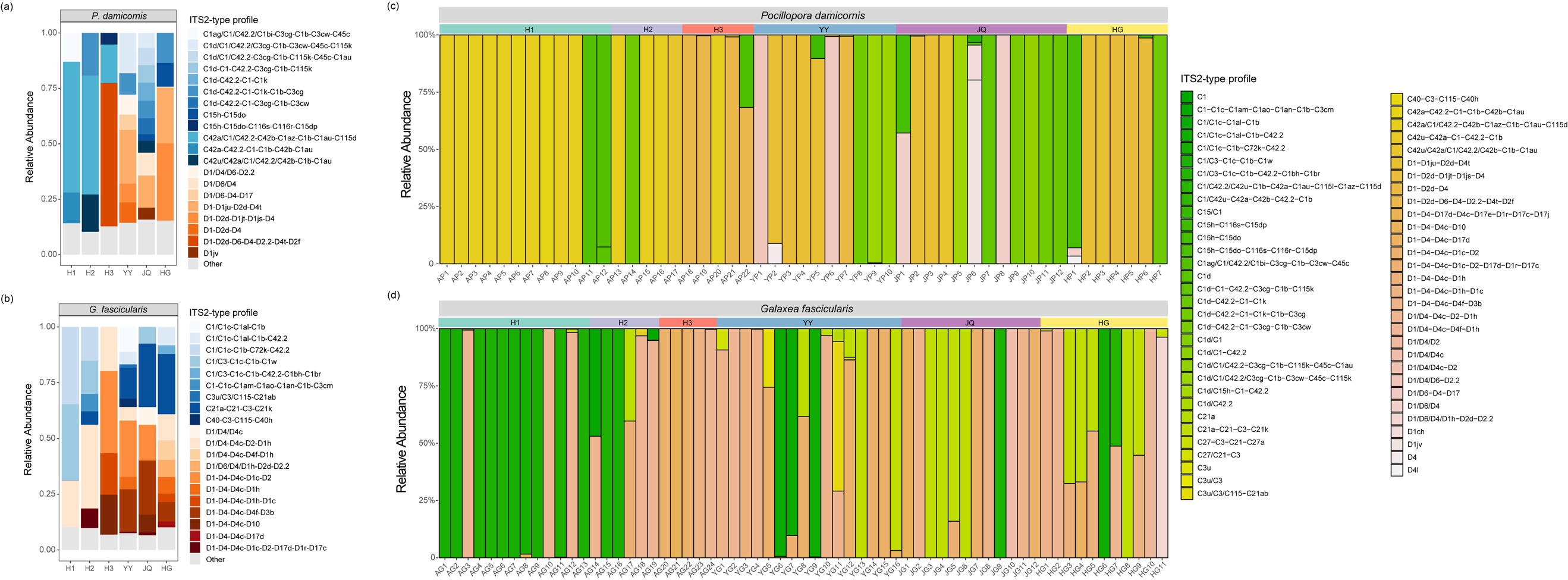


Figure S3. Relative abundances of the 20 most abundant SymPortal Symbiodiniaceae ITS2-type profiles in *Pocillopora damicornis* (a) and *Galaxea fascicularis* (b). Normalized relative abundance of 62 Symbiodiniaceae ITS2-type profiles from *P. damicornis* (c) and *G. fascicularis* (d).


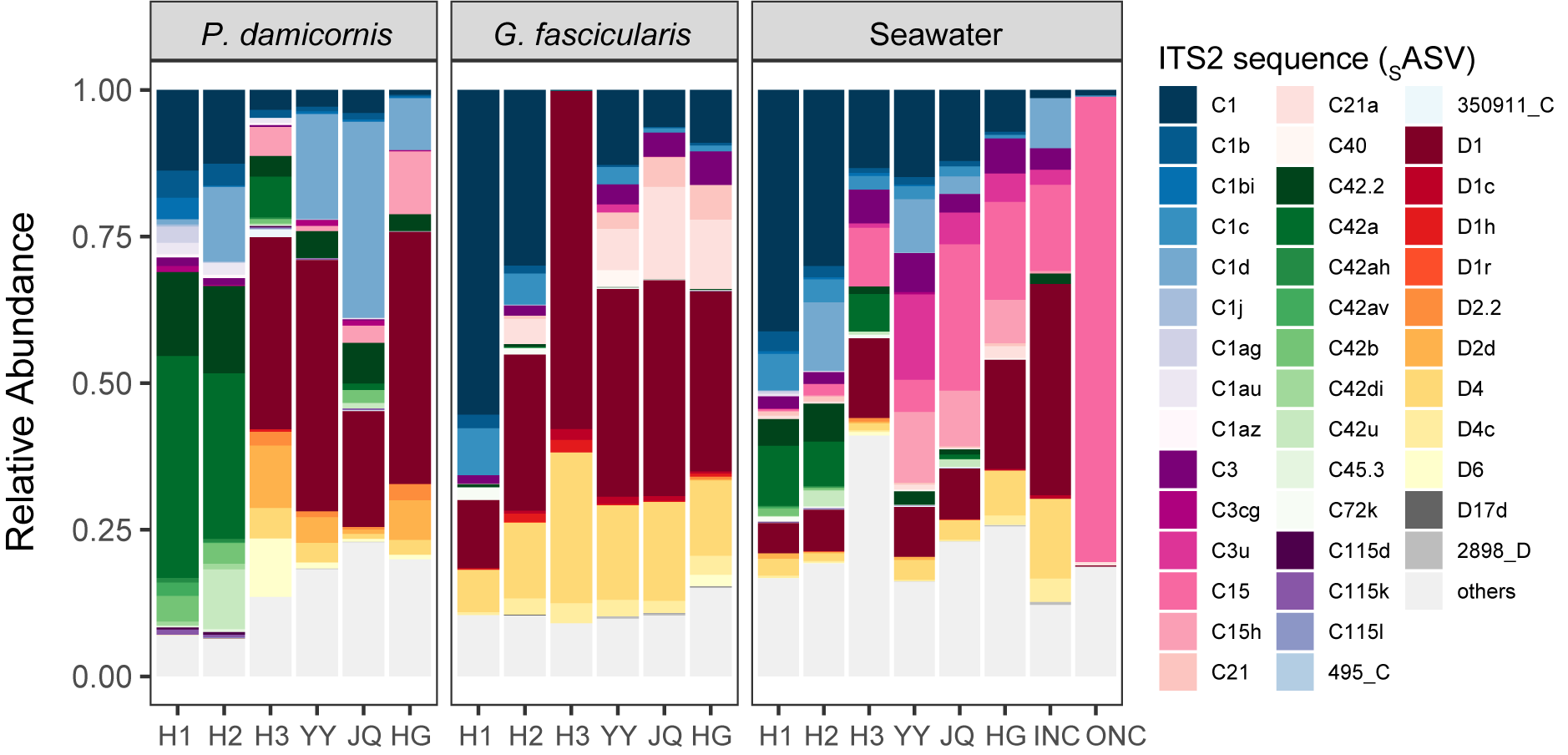


Figure S4. Relative abundances of the 42 shared _S_ASVs in different habitats.
